# Serotonin-1A receptor alterations in depression: a meta-analysis of molecular imaging studies

**DOI:** 10.1186/s12888-016-1025-0

**Published:** 2016-09-13

**Authors:** Ling Wang, Chanjuan Zhou, Dan Zhu, Xinfa Wang, Liang Fang, Jiaju Zhong, Qiang Mao, Lu Sun, Xue Gong, Jinjun Xia, Bing Lian, Peng Xie

**Affiliations:** 1 Chongqing Key Laboratory of Neurobiology, Chongqing Medical University, Yixueyuan Road, Yuzhong District Chongqing, China; 2 Institute of Neuroscience and the Collaborative Innovation Center for Brain Science, Chongqing Medical University, Yixueyuan Road, Yuzhong District Chongqing, China; 3 Department of Neurology, The First Affiliated Hospital of Chongqing Medical University, Youyi Road, Yuzhong District Chongqing, China; 4 Institute of Neuroscience, Chongqing Medical University, Yixueyuan Road, Yuzhong District Chongqing, China; 5 Department of Neurology, The Yongchuan Hospital of Chongqing Medical University, Xuanhua Road, Yongchuan District Chongqing, China

**Keywords:** Meta-analysis, 5-HT_1A_, Molecular imaging, Depression

## Abstract

**Background:**

Postmortem studies of people who have successfully committed suicide and people with depression have implicated the serotonin-1A (5-HT_1A_) receptor system in the pathophysiology of depression. Several molecular imaging studies have investigated alterations in 5-HT_1A_ receptors in patients with depression using positron emission tomography and have reported conflicting results.

**Methods:**

We performed a meta-analysis of studies investigating the relationship between depression and 5-HT_1A_ binding. We conducted a comprehensive search of Medline, Embase, ScienceDirect, Scopus and Springer databases for relevant studies published between January 1999 and October 2015. The meta-analysis was conducted in accordance with the Meta-analysis of Observational Studies in Epidemiology guidelines.

**Results:**

Ten studies were included, comprising 218 patients with depression and 261 healthy controls. The results of these studies indicated a reduction in 5-HT_1A_ receptors in mesiotemporal cortex, yielding a summary effect estimate of -0.8 (95 % CI −1.36, −0.24). Smaller reductions were reported in 5-HT_1A_ receptor binding in the hippocampus, raphe nuclei, insular, anterior cingulate cortex and occipital cortex of people with depression. No clear effect of depression on 5-HT_1A_ receptors was detected in the amygdala.

**Conclusions:**

Reduced 5-HT_1A_ receptor binding was associated with the pathology of depression and predicted altered serotonergic neurotransmission in various brain regions. These findings increase our understanding of the neurophysiological processes underlying depression.

**Electronic supplementary material:**

The online version of this article (doi:10.1186/s12888-016-1025-0) contains supplementary material, which is available to authorized users.

## Background

Depression is a chronic mental illness characterized by depressed mood, anhedonia, irritability, concentration difficulties, and abnormalities in appetite and sleep. It has a lifetime prevalence of 10–15 % [21]. The classic biogenic amine hypothesis of depression suggests that the disorder is associated with a deficiency in several neurotransmitters, including serotonin (5-hydroxytryptamine, 5-HT), noradrenaline (NA), and acetylcholine (ACh) [3]. There is increasing evidence that alterations in the brain serotonergic system are involved in the pathophysiology of depression [13, 42]. It has been suggested that 5-HT receptor dysfunction might contribute significantly to the development of depression.

5-HT receptors are highly expressed in the human limbic system, including the amygdala, hippocampus, thalamus, putamen, anterior cingulate cortex and midbrain [37]. Among the 5-HT receptor types (5-HT_1A_, 5-HT_1B_, 5-HT_2A_, and 5-HT_4_), 5-HT_1A_ has generated much research interest because of its involvement in recognition, learning memory, and hippocampal neurogenesis, as well as its response to antidepressant treatment [15, 36, 41]. Furthermore, 5-HT_1A_ dysfunction often accompanies depression. Extensive rodent and human research suggests that 5-HT_1A_ dysregulation is highly sensitive to stress, i.e., increased cortisol levels [9, 25].

A previous meta-analysis of molecular imaging studies of depression reported a relationship between the disorder and serotonin transporters, which indicated altered serotonergic availability in depression [14]. Recent functional neuroimaging has pointed to widespread abnormalities in 5-HT_1A_ binding in depression. Since 1991, abnormalities in 5-HT_1A_ binding in patients with depression have been investigated using positron emission tomography (PET) techniques under various scanning conditions [1]. Several such studies have reported decreased 5-HT_1A_ binding in patients with depression compared with healthy controls [34, 38]. However, other studies have reported conflicting results or insufficient evidence to confirm any relationship [20, 22, 23, 31].

While converging lines of evidence indicate that 5-HT_1A_ may contribute to the pathophysiology of depression, there is no consensus about the way 5-HT_1A_ binding is altered in the condition. To address this contention, we performed a meta-analysis of studies investigating the relationship between depression and 5-HT_1A_ binding. We hypothesized that at least some of the discrepancies between studies would be explained by our meta-analysis.

## Methods

To ensure the quality of this meta-analysis, we followed the proposal for conducting and reporting described in ‘Meta-analysis of Observational Studies in Epidemiology (MOOSE)’ (Stroup DF 2000 [47]). The MOOSE checklist is included in the Additional file 1.

### Search strategy

Two reviewers (LW and CjZ), one postgraduate student and one doctoral student, systematically searched Medline, Embase, ScienceDirect, Scopus, and Springer databases to identify relevant manuscripts published between January 1999 and October 2015. Databases were accessed via PubMed or directly via their website.

We used subject and free-form search terms as follows (the PubMed search string is provided as an example):

- #1 depression [MeSH Terms] OR depress* OR bipolar disorder OR affective disorders, psychotic OR major depression;
- #2 positron-Emission tomography [MeSH Terms] OR pet OR tomography, emission-computed, single-photon OR SPECT OR molecular imaging OR molecular diagno*;
- #3 receptor, serotonin, 5-HT1A[MeSH Terms] OR serotonin 1A receptor;
- #4 #1 AND #2 AND #3.

### Study selection and data extraction

The inclusion criteria were: (1) original studies that indexed 5-HT_1A_ receptors in patients with depression and healthy controls; (2) molecular imaging studies published in English. We excluded: (1) subjects with neurological, severe somatic, psychotic, chronic stress or affective disorders other than unipolar depression or bipolar depression; (2) reviews, comments, and case reports; (3) overlapping or duplicated samples; (4) subjects with depression accompanied by neuropsychiatric or physical diseases; and (5) studies that included relatives with significant symptoms. Where several contrasts or tasks were present in the same study, we chose one task to avoid including the sample twice.

The binding potential (BP) value of [11C]WAY-100635 was used as the primary outcome for the analysis. This radioligand binds specifically to 5-HT_1A_ and is widely used in PET studies to measure 5-HT_1A_ occupancy and density in psychiatric patients [12]. Secondary outcomes (listed by relevance to clinical and population characteristics) included: diagnosis, symptoms severity, diagnosis criteria for psychiatric diagnosis, rating tools, antidepressant treatment, study and patient characteristics, measurements performed, year of publication, population characteristics, and type of tracer. Two reviewers (LW and CjZ) extracted all data independently. Any disagreement between reviewers was resolved by discussion.

### Statistical methods

Statistical analyses were performed using RevMan version 5.0.1 (The Cochrane Collaboration The Nordic Cochrane Centre, Copenhagen, Denmark). The BP value in given regions was weighted by standard mean differences (SMD) with a 95 % confidence interval (CI) for each individual study. We used Q-tests with a significance threshold of *p* < 0.05 (two-tailed) to evaluate the SMD.

We assessed heterogeneity using a chi-squared Q-statistic, and its magnitude was estimated using the inconsistency index I^2^. I^2^ indicates the percentage of effect size variance due to heterogeneity: I^2^ = 100 % × (Q-df)/Q [16]. If between-study variance clearly made the assessment of heterogeneity significant (*p <* 0.05), a random-effects model was used to estimate the effects of major depressive disorder (MDD) on 5-HT_1A_ expression.

Publication bias is the tendency of small studies to report large effect sizes. We assessed this parameter using Begg’s funnel plots. The presence of bias was indicated by asymmetrical plots. In addition, we conducted a sensitivity analysis by excluding studies that might influence pooled SMD.

## Results

### Search results

**Fig. 1:**
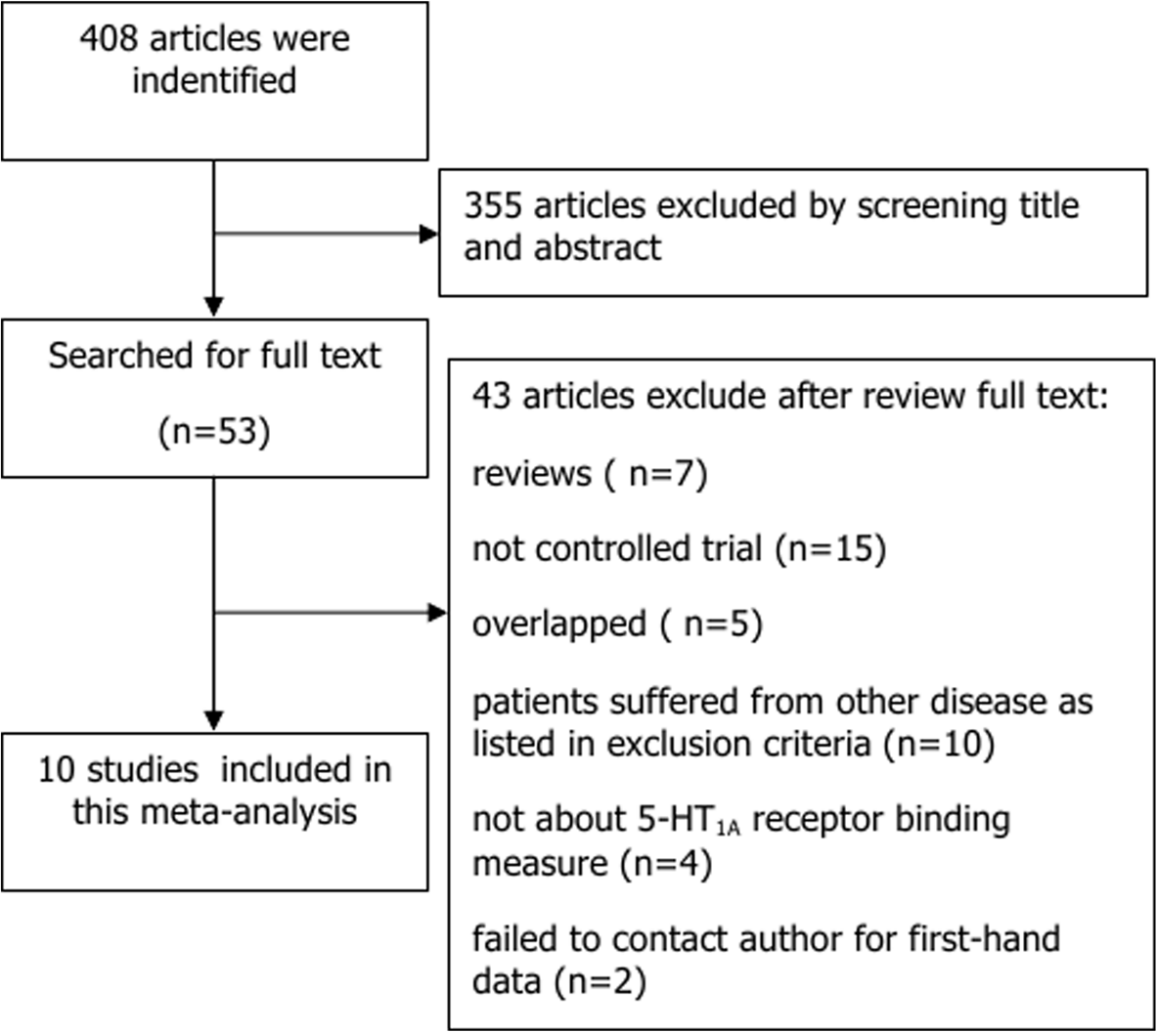
Flowchart of identification, screening and inclusion of eligible studies

We identified 408 potentially relevant studies, which were managed in Endnote X7 (Thomas Reuters Scientific, Philadelphia, PA, USA). After scanning each title and abstract, 355 studies were excluded. Of the 53 remaining articles, 43 were further excluded: seven articles were systematic reviews; four articles did not report 5-HT_1A_ binding measures; 15 articles were not controlled clinical trials; ten articles included patients with depression accompanied by neuropsychiatric or physical diseases, or chronic stress or affective disorders other than unipolar depression or bipolar depression; two articles did not supply adequate data; and five articles were molecular imaging studies that likely overlapped with another included study. Finally, ten studies remained for inclusion in the meta-analysis ([4, 11, 18, 28, 32, 33, 35, 39, 43, 48]) (see Fig. 1). Three of the included studies (marked with a “Δ” in Table 1) did not provide quantitative data, and data were only available in a graphical format. For these studies, where we could not obtain primary data by contacting the author, we used GetData Graph Digitizer version 2.2 (GetData, Moscow, Russia) to analyze the graphical data.

**Table 1:** Key characteristics of selected studies

1st author	Publication year	Method/Ligand	Reference region	Diagnosis	Severity ranting tools	Severity score	Controls (F) + age	Patients (F) + age	Treatment
Wayne C. Drevets	1999	PET [11C]WAY-100635	Cerebellum	MDD, BPII	HAM-17	22 ± 6.4	8 (4) 35.3 ± 13.5	12 (7) 35.8 ± 9.7	Drug free ≥2 week
Sargent P. A^a^.	2000	PET [11C]WAY-100635	Cerebellum	MDD, BPII	HAM-17	14.1 ± 194.9	18 (1) 36.4 ± 8.3	35 (3) 40 ± 34.5	Drug free ≥12
Z Bhagwagar	2004	PET [11C]WAY-100635	Cerebellum	MDD	HAM-7	2.3 ± 1	18 (0) 43.2 ± 13	14 (0) 48 ± 14.9	Drug free ≥25.7 week
Carolyn Cidis Meltzer	2004	PET [11C]WAY-100635	Plasma	MDD	HAM-17	18.1 ± 2.7	17 (9) 70 ± 6.7	17 (13) 71.4 ± 5.9	Drug free ≥2 week
Jussi Hirvonen△	2008	PET [11C]WAY-100635	Plasma	MDD	HAM-17	18.1 ± 2.9	15 (8) 32.6 ± 7.7	21 (8) 40.1 ± 9	Drug free ≥17.1 week
Eydie L. Moses-kolko	2008	PET [11C]WAY-100635	Cerebellum	Postpartum D.	HAM-17	21 ± 4.3	7 (7) 33 ± 3.9	9 (9) 26.9 ± 7.9	7 patients rdrug naïve
Ramin V. Parsey ^a^△	2006b	PET [11C]WAY-100635	Cerebellum	MDD	HAM-17	25.7 ± 7.08	43 (24) 38.2 ± 15	28 (7) 38.5 ± 29.3	Drug free ≥2 week
Gregory M. Sullivan△	2009	PET [11C]WAY-100635	Cerebellar white matter	BPII	HAM-17	18 ± 4.9	47 (27) 38.1 ± 14.7	32 (19) 38.4 ± 9.7	Drug free ≥2 week
Jeffrey Miller^a^△	2012	PET [11C]WAY-100635	Cerebellum white matter	MDD	HAM-17	24.6 ± 5.3	51 (29) 37.3 ± 14.4	24 (17) 35 ± 13.3	Drug free ≥3
Allison C. Nugent	2013a	PET [11C]WAY-100635	Cerebellar white matte	BPII	MADRS	23 ± 10	33 ± 9.4	26 (19) 33 ± 9.5	Drug free ≥3 week

*MDD* major depressive disorder, *BP* bipolar depression, *PET* positron emission tomography, *HAMD* Hamilton depression scale, *MADRS* Montgomery and Asberg depression rating scale

^a^Data from subgroups were combined and averaged (severity score, age, BP values)

ΔBP values were acquired from figures by graphical software (GetData Graph Digitizer v2.2)

Table 1 shows data extracted from the ten included articles, comprising 218 patients with depression and 261 healthy controls. The most frequently studied brain regions in studies using PET to measure 5-HT_1A_ binding were the limbic system (cingulate cortex, amygdala, hippocampus/parahippocampal), cortical regions (occipital cortex, temporal cortex, prefrontal cortex), raphe nuclei, and mesiotemporal cortex. In this meta-analysis, to ensure reliability of findings, regions were included if they were reported in a minimum of four studies. If sub-regions within a structure (e.g. left mesiotemporal cortex and right mesiotemporal cortex) were reported, the corresponding 5-HT_1A_ BP values were combined and averaged, according to the Cochrane Handbook for Systematic Reviews of Interventions version 5.0 [17].

### Meta-analysis of 5-HT_1A_ binding in depression

This meta-analysis focused on differences in 5-HT_1A_ binding between patients with depression and healthy controls in six reported regions: hippocampus (HIP), mesiotemporal cortex (MTC), anterior cingulate cortex (ACN), occipital cortex (OCC), raphe nucleus (RN), and insular cortex (INS).

**Fig. 2:**
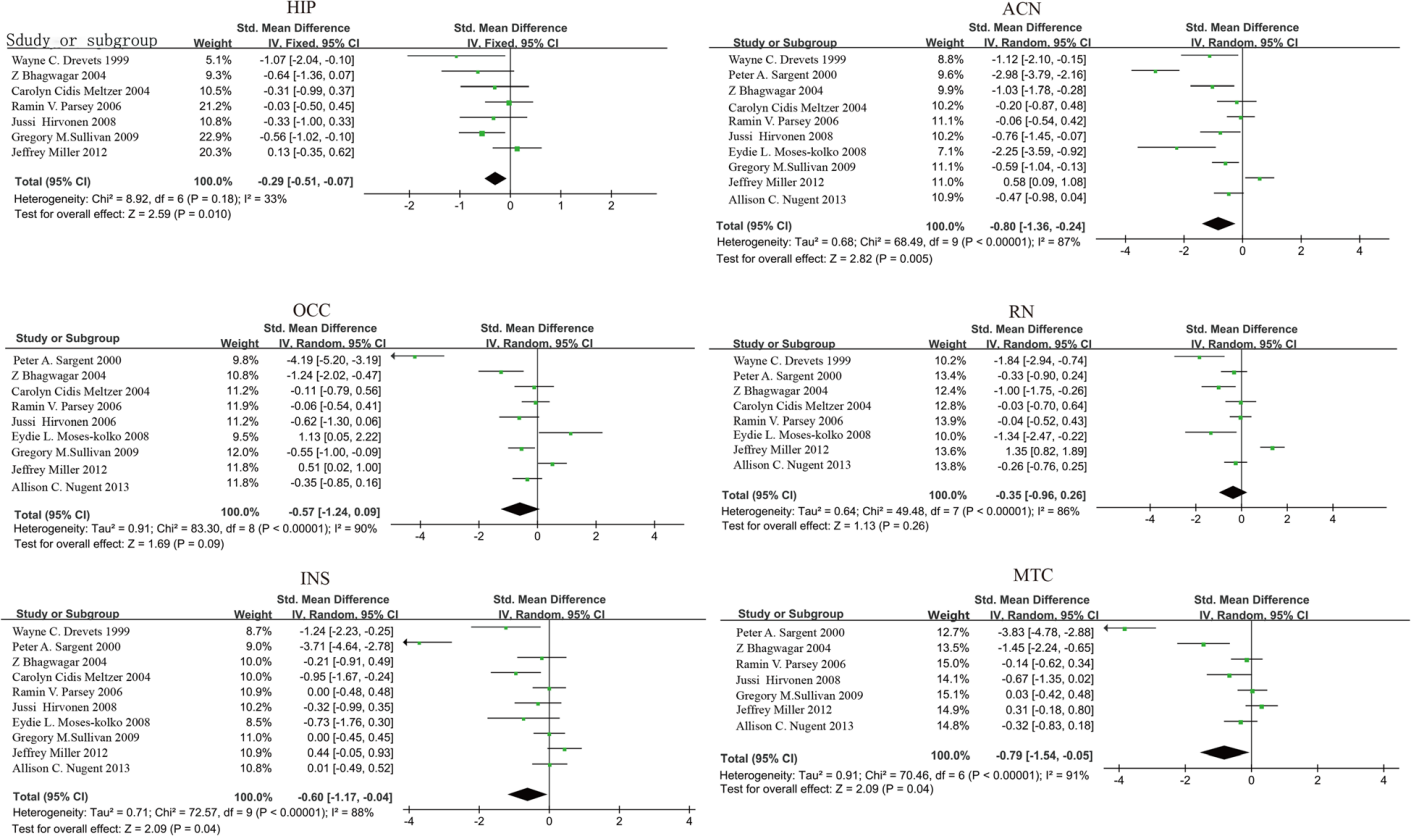
Forest plots showing the summary effect sizes of 5-HT_1A_ binding in depression. HIP: hippocampus; MTC: mesiotemporal cortex; CAN: anterior cingulate cortex; OCC: occipital cortex; RN: raphe nucleus; INS: insular cortex

Ten included studies investigated 5-HT_1A_ binding in MTC. The analysis yielded significantly lower binding in people with depression, with an effect estimate of −0.8 (95 % CI −1.36, −0.24). Additionally, a moderate reduction of 5-HT_1A_ binding in people with depression relative to healthy controls was detected in HIP: −0.29 (95 % CI −0.51, −0.07), CAN: −0.57 (95 % CI −1.24, −0.09), OCC: −0.35 (95 % CI −0.96, −0.04), RN: −0.60 (95 % CI −1.17, −0.04) and INS: −0.79 (95 % CI −0.54, −0.05) (Fig. 2).

In some studies, differences in 5-HT_1A_ binding were reported between patients with and without remission. Therefore, we assumed that the severity of depressive symptoms might influence 5-HT_1A_ availability. However, no relationship was found between depressive symptom severity and 5-HT_1A_ availability following subgroup analysis in the six regions mentioned above.

### Sensitivity analysis

We hypothesized that depressive symptom severity, small sample size (each arm containing fewer than 15 patients), and the chosen reference tissue might influence 5-HT_1A_ availability measures (Additional files 1, 2 and 3). To test this hypothesis, we performed several sensitivity analyses by deleting studies with each factor respectively to assess which factors influenced the results.

The results indicated that overall significance of the SMD was altered after exclusion of two studies with small sample sizes and one study with conflicting results [32, 33, 43]. The results following exclusion of these studies were as follows: MTC: −0.49 (95 % CI −0.71, −0.27), *p* = 0.0001 for Q test, I^2^ = 27; HIP: −0.40 (95 % CI −0.64, −0.19), *p* = 0.002 for Q test, I^2^ = 5; CAN: −0.41 (95 % CI −0.64, −0.19), *p* = 0.0004 for Q test, I^2^ = 36; OCC: −0.50 (95 % CI −1.02, −0.02), *p* = 0.01 for Q test, I^2^ = 68; RN: −0.21 (95 % CI −0.43, −0.02), *p* = 0.07 for Q test, I^2^ = 44; and INS: −0.43 (95 % CI −0.86, −0.00), *p* = 0.009 for Q test, I^2^ = 66. These changes suggest that the findings of the meta-analysis were strongly influenced by these three studies.

### Publication bias

**Fig. 3:**
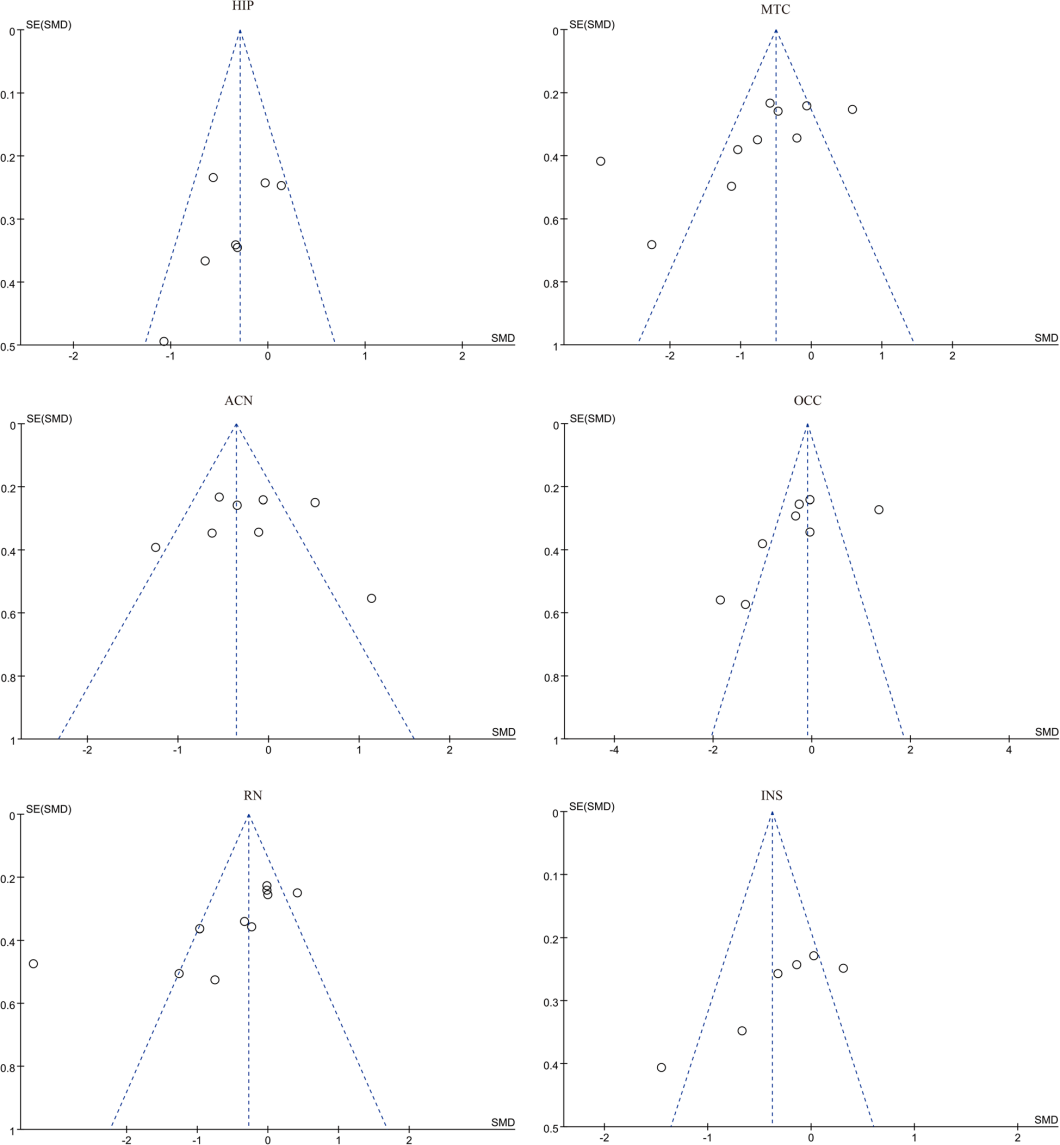
Funnel plot analyzing publication bias for the summary effect sizes of 5-HT_1A_ binding in depression. HIP: hippocampus; MTC: mesiotemporal cortex; CAN: anterior cingulate cortex; OCC: occipital cortex; RN: raphe nucleus; INS: insular cortex

A funnel plot was generated to explore publication bias (Fig. 3). The results indicated that some publication bias was present in the analyses of MTC, CAN, INS, OCC, and RN. Publication bias may have been due to the limited number (*n* ≤ 10) of studies we included. There was no evidence for publication bias in HIP.

## Discussion

### 5-HT_1A_ changes in depression

Our literature search yielded ten published studies of 5-HT_1A_ receptor binding in depression, comprising 218 patients with depression and 261 healthy controls. We found a mean group size of 22 patients and 26 controls per imaging study. Our meta-analysis indicated significantly decreased 5-HT_1A_ density in MTC, and smaller reductions in 5-HT_1A_ binding in RN, INS, HIP, CAN and OCC in patients with depression. The different PET imaging methods employed in each study to measure 5-HT_1A_ binding in high-affinity regions (i.e., RN, INS, and OCC) may have contributed to high inter-study variability for these particular regions.

Some previous studies have reported that 5-HT_1A_ alterations in depression are influenced by antidepressant medication, and remission and recovery status. In the 10 studies we included, patients were prescribed antidepressants prior to study enrollment, but details of drug doses and types were not reported. It was therefore difficult to investigate whether antidepressant medication affected 5-HT_1A_ availability during treatment in our meta-analysis. We hypothesized that 5-HT_1A_ binding would be associated with depressive symptom severity. However, no relationship was found between the severity of depressive symptoms and 5-HT_1A_ availability in patients with depression.

Reduced 5-HT_1A_ binding visualized with PET may reflect changes in receptor density or affinity. Alternatively, such reductions may reflect receptor down-regulation, inter-activation, or blockage by endogenous ligands (although [11C] WAY-100635 appears to be insensitive to endogenous levels of serotonin; [6]). In the current meta-analysis, we found significant reductions in 5-HT_1A_ in MTC (including amygdala). This finding is consistent with previous postmortem studies indicating lower 5-HT_1A_ binding and mRNA expression in MDD and bipolar disorder [24, 26, 27]. Postsynaptic 5-HT_1A_ receptors exist in cortical inter-neurons and pyramidal dendrites. These receptors participate in feedback inhibition of 5-HT neuronal activity and the modulation of cortical circuits [37]. Decreased 5-HT_1A_ binding may act as a compensating factor to improve postsynaptic serotonin reuptake during a depressive episode. We speculate that decreased binding in the MTC of people with depression may impair the integration of 5-HT signaling in cortico-mesiotemporal cortical circuits and disrupt limbic input back to the cortex [2, 40]. Thus, the reduction of 5-HT_1A_ binding in depression found in our meta-analysis may represent mesiotemporal 5-HT_1A_-mediated dysregulation of cortical and limbic structures.

We detected smaller differences in 5-HT_1A_ binding between depression and healthy controls in HIP, RN, CAN, and OCC. However, sensitivity analyses revealed stronger trends toward reduced 5-HT_1A_ binding. The HIP has previously been shown to be structurally and functionally altered during depression [8, 29, 30]. However, the data regarding 5-HT_1A_ binding in OCC and RN merit special attention for several reasons. First, there is evidence that the RN contains many serotonergic cell bodies that regulate 5-HT release and uptake. Collin et al. [10] found that the level of expression of 5-HT transporter mRNA was down-regulated in the rodent dorsal raphe nucleus in obese behaviorally depressed ob/ob mice. Decreased 5-HT_1A_ autoreceptor binding was also found in the dorsal raphe nucleus of people with depression who successfully committed suicide [7]. Moreover, several studies have indicated that the OCC is involved in depression, and γ-aminobutyric acid (GABA) concentrations in this region have been shown to be functionally altered in people with depression [5, 44]. These data support the hypothesis that depression is associated with reduced presynaptic serotonergic activity, which leads to the down-regulation of postsynaptic 5-HT_1A_ sites. The results of the current meta-analysis suggest that further imaging and animal studies are warranted to investigate the specific role of 5-HT_1A_ and serotonergic dysfunction in the OCC and RN of patients with depression.

### Study heterogeneity

Study heterogeneity occurs when multiple studies investigating a particular effect are actually measuring different effects. This may be caused by differences in samples, interventions, statistical analyses, or study designs, and may cause unreliability in meta-analyses. In the current study, several factors may have caused high heterogeneity, including the severity of depressive symptoms, small sample sizes, and the chosen reference tissue for normalization in PET. Differences in PET scanning protocols were also considered to be potentially important sources of variation between measurements [45]. Our results indicate that considerably more data are required for regions with high 5-HT_1A_ abundance. Moreover, we found that the regions chosen for normalization of specific binding to the radioactivity concentration in a reference region differed between studies (plasma, cerebellum gray matter, cerebellum white matter, whole cerebellum). These limitations, in combination with the measurement error and substantial biological variability in 5-HT_1A_, may have caused instability in group differences, reflecting an inherently problematic aspect of data acquisition using PET. These findings highlight the ways that specific technical differences in data collection and analysis can produce conflicting or inconsistent results. The development of a gold standard for arterial blood with larger sample sizes in PET studies may provide a solution that enables definitive conclusions about 5-HT_1A_ density in depression.

The association between the C(-1019)G polymorphism in the promoter region of the 5-HT_1A_ gene and depression may be another relevant factor. There is evidence that higher expression of the G allele may increase the risk of developing depression [19, 46, 49]. Therefore, genetic variability may contribute to heterogeneity in studies of 5-HT_1A_ receptors in depression. It is possible that the inter-study heterogeneity in SMD in the current results was caused by genetic variation in 5-HT_1A_ receptors. However, we did not consider genetic variation in our meta-analysis.

### Study limitations

Several limitations should be considered in the interpretation of our findings. First, the number of published studies included was too small to exclude small study bias (i.e., smaller studies contributing to larger effect sizes). This finding indicates that future clinical molecular imaging studies should include larger sample sizes. Second, some brain regions have been associated with altered 5-HT_1A_ binding but were not included in the current analysis because of an insufficient number of studies reporting data for these regions. Third, we suspect that heterogeneity may result from genetic variation, as well as differences in gender, depression severity, and treatment. However, we did not analyze these factors, and did not obtain sufficiently detailed data to allow such an analysis. Finally, we detected publication bias in this meta-analysis, possibly resulting from the small number of studies included.

## Conclusions

To our knowledge, this is the first meta-analysis of molecular imaging studies of 5-HT_1A_ binding in depression. It has been widely assumed that depression is associated with changes in the 5-HT system. However, consistent evidence from molecular imaging studies is limited. To resolve this uncertainty, we performed a systematic review and meta-analysis, which yielded ten molecular imaging studies of depression. Our meta-analysis showed a decrease in 5-HT_1A_ binding in MTC associated with depression. Smaller reductions in 5-HT_1A_ binding in HIP, RN, CAN, OCC, and INS of patients with depression were also found.

We conclude that individual molecular imaging studies have lacked sufficient statistical power to detect serotonergic dysfunctions in depression. Furthermore, potentially relevant factors, such as sample size, scanning protocol, and genetic polymorphisms, should be considered in future PET studies to further elucidate the pathophysiological mechanisms underlying depression.

## Additional files

Additional file 1: MOOSE Checklist. A list of factors that have been checked or done for this meta-analysis of observational studies. (DOC 77 kb) Additional file 2: Primary Data. Data extracted from the articles that met the inclusion criteria. (XLSX 25 kb) Additional file 3: Study Quality Assessment. The quality assessment for each article included. (XLSX 16 kb)
